# Indoleamine 2, 3-dioxygenase is responsible for low stress tolerance after intracerebral hemorrhage

**DOI:** 10.1371/journal.pone.0273037

**Published:** 2023-02-08

**Authors:** Masatoshi Ohnishi, Marina Akagi, Mako Kotsuki, Seishi Yonemura, Hikari Aokawa, Maki Yamashita-Ibara, Osamu Yokofujita, Shoji Maehara, Toshiyuki Hata, Atsuko Inoue

**Affiliations:** 1 Department of Pharmacotherapeutics, Graduate School of Pharmacy and Pharmaceutical Sciences, Fukuyama University, Hiroshima, Japan; 2 Department of Pharmacotherapeutics, Faculty of Pharmacy and Pharmaceutical Sciences, Fukuyama University, Hiroshima, Japan; 3 Department of Physical Chemistry for Bioactive Molecules, Faculty of Pharmacy and Pharmaceutical Sciences, Fukuyama University, Hiroshima, Japan; Universita degli Studi di Napoli Federico II, ITALY

## Abstract

In the chronic phase after intracerebral hemorrhage (ICH), the aftereffect-associated lowering of motivation burdens many patients; however, the pathogenic mechanism is unclear. Here, we revealed for the first time that indoleamine 2, 3-dioxygenase (IDO) expression and enzyme activity are increased in the collagenase-induced murine ICH model. IDO is a rate-limiting enzyme situated at the beginning of the kynurenine pathway and converts tryptophan, a source of serotonin (5-hydroxytryptamine; 5-HT), to kynurenine. In this study, we showed that IDO is localized in 5-HTergic neurons. After ICH, the synaptosomal 5-HT level decreased, but this effect was neutralized by subcutaneous injections of 1-methyl tryptophan (MT), a specific IDO inhibitor. These results suggest that ICH-induced IDO weakens the activity of 5-HTergic neurons. Accordingly, we next investigated whether the IDO increase contributes to the depression-like behaviors of ICH mice. The immobility times of tail suspension and forced swimming tests were significantly prolonged after ICH but shortened by the administration of 1-MT. In conclusion, the increased IDO after ICH was found to decrease 5-HT levels and subsequently reduce stress tolerance. These findings indicate that IDO is a novel therapeutic target for the ICH aftereffect-associated lowering of motivation.

## Introduction

Intracerebral hemorrhage (ICH) is a type of hemorrhagic stroke and is mainly caused by hypertension and vascular malformation. Many ICH patients experience the lowering of motivation as an aftereffect, and this secondary depression-like symptom often impairs the course of recovery. Depression follows cerebral ischemia in approximately one-third of patients [1]; however, there is little literature about this depression.

Indoleamine 2, 3-dioxygenase (IDO) is a rate-limiting enzyme situated at the first step of the kynurenine pathway, and this enzyme converts tryptophan to nicotinamide [2]. IDO reduces tryptophan, a source of serotonin (5-hydroxytryptamine; 5-HT), and has been reported to also reduce 5-HT itself [3]. Serotonin is well known as a bioactive amine that stabilizes mood. In this context, emerging evidence support that IDO contributes to various psychiatric symptoms. For example, one potential role of IDO that has been suggested for a long time is the involvement in neuropsychiatric symptoms during interferon (IFN) therapy [4, 5]. The kynurenine pathway produces several intermediary metabolites such as quinolinic acid, kynurenic acid, and 3-hydroxykynurenine. Among these metabolites, the levels of quinolinic acid and kynurenine in the spinal fluid of ICH child patients were reported to be markedly increased [6], suggestive of increased IDO expression in their brains; however, there is no direct evidence of this increase. IDO is basally expressed in neurons [7, 8]. Although IDO expression is thought to transiently increase in hippocampal neurons after ischemic stroke [9, 10], it is unclear whether this event contributes to the depression aftereffect.

Accordingly, we hypothesized that an increase in IDO after ICH contributes to the aftereffect-associated lowering of motivation. We therefore investigated changes in IDO expression levels after ICH and the effect of an IDO inhibitor on brain 5-HT levels and on depression-like behavior.

## Materials and methods

### Drugs and chemicals

All drugs and chemicals were purchased from Nacalai Tesque (Kyoto, Japan) unless otherwise indicated. Collagenase type VII and 1-methyl tryptophan (MT) were obtained from Sigma-Aldrich (St. Louis, MO, USA).

### Preparation of the *in vivo* murine ICH model and drug administration

The methods in the present study were approved by the Institutional Animal Care and Use Committee of Fukuyama University (H26-A-5, 2019-A-5), and the animals were treated in accordance with the guidelines of the United States National Institutes of Health regarding the care and use of animals for experimental procedures.

In total, 292 male ddY mice (Nihon SLC, Shizuoka, Japan) were used. Mice were housed at 22 ± 1°C under a 12-h light and dark cycle with free access to food and water. The mice were anesthetized by intraperitoneal injection of a mixture of medetomidine (0.3 mg/kg, Kyoritsu Seiyaku, Tokyo, Japan), midazolam (4 mg/kg, Alfresa, Osaka, Japan), and butorphanol tartrate (5 mg/kg, Meiji Seika Pharma, Tokyo, Japan), and then placed in a stereotaxic frame (Narishige, Tokyo, Japan). Collagenase type VII (0.03 U/3 μL saline) or the same amount of saline was injected via a Hamilton syringe into the unilateral striatum (0.2 mm anterior, 2.2 mm lateral from the bregma suture, and 3.5 mm depth from the skull) for 3 min [11]. The syringe was slowly removed 1 min after the injection, and then the scalp incision was sutured. An IDO inhibitor, 1-MT (1 mg), was dissolved in saline using 0.1 N NaOH solution and injected subcutaneously 1 h after collagenase injection and then daily. Control mice were injected with saline instead of 1-MT.

### Measurement of hematoma size

Brains were extirpated and cut into coronal sections containing a syringe mask for collagenase injection using a brain slicer (Muromachi Kikai, Tokyo, Japan). Hematoma size was estimated from the hematoma area in the cross-sections using ImageJ [11, 12].

### Real-time PCR

Total RNA was obtained from the striatal tissues collected from each mouse according to the acid guanidinium thiocyanate-phenol-chloroform method [13]. Complementary DNA was obtained using 4 μg total RNA, 50 U M-MLV Reverse Transcriptase (Thermo Fisher Scientific, Waltham, MA, USA), and 50 pmol random primers, and subjected to quantitative PCR using a LightCycler (Roche, Basel, Switzerland). The amount of cDNA corresponding to 0.1 μg of original total RNA was used per reaction with the LightCycler Fast Start DNA Master SYBR Green I (Roche) and 1 μM gene-specific primers. Primer sequences used in the study are shown in Table 1. The amounts of sample mRNA were set relative to the control group using LightCycler software (Roche) and expressed as values normalized to the GAPDH level.

**Table 1:** Primers for real-time PCR.

	Forward	Reverse
IDO	5’-CCTGCCTCCTATTCTGTC-3’	5’-CTGGTAGCTATGTCGTGC-3’
GAPDH	5’-CTTCCACCACCATGGAGAA-3’	5’-AAGCAGTTGGTGGTGCAG-3’

### Western blotting

Perihematomal tissues were homogenized with lysis buffer (0.2 M Tris (pH 7.0), 250 mM β-glycerophosphate, 10 mM ethylene glycol tetraacetic acid-2Na, and 10% Triton-X). After centrifugation, Laemmli buffer (124 mM Tris-HCl (pH 6.8), 4% sodium dodecyl sulfate (SDS), 10% glycerol, 0.02% bromophenol blue, and 4% 2-mercaptoethanol) was added to the supernatants, and the mixtures were boiled for 5 min. The samples were subjected to 12% SDS polyacrylamide gel electrophoresis and then transferred to nitrocellulose membranes (GE Healthcare, Boston, MA, USA) for 60 min. The membranes were blocked with 5% skim milk (Morinaga, Tokyo, Japan) for 60 min at room temperature and then incubated with mouse anti-IDO (1:500) (Chemicon, Tokyo, Japan) and mouse anti-β-actin (1:1000) (Santa Cruz Biotechnology, Santa Cruz, CA, USA) antibodies overnight at 4°C. Finally, the membranes were rinsed and incubated with ECL anti-mouse IgG horseradish peroxidase-linked whole antibody from sheep (1:10000) (GE Healthcare) for 60 min at room temperature. After rinsing, secondary antibodies were detected with ECL western blotting detection reagents (GE Healthcare) for 1 min. The amounts of protein were analyzed using a CS Analyzer (Atto, Tokyo, Japan) and defined relative to the control group as values normalized to β-actin.

### Immunohistochemistry

Mice were deeply anesthetized, transcardially perfused, and fixed with 10 mL ice-cold phosphate-buffered saline (PBS) followed by 4% paraformaldehyde (PFA). The whole brains were dissected out, further fixed in 4% PFA for 60 min, and soaked in 15% sucrose solution until completely sinking at 4°C. The cerebellums were removed from the whole brains, and cerebra were frozen in dry ice and then in a -80°C deep freezer (Sanyo, Tokyo, Japan). Specimens containing the striatum area were cut into 10-μm thick sections using a Cryostat (Leica, Wetzlar, Germany) and placed on CREST-coated slide glasses (Matsunami Glass, Osaka, Japan). After rinsing with PBS, the sections were blocked and permeabilized with 5% goat serum/0.5% Triton X-100 in PBS for 60 min at room temperature. Then, the sections were incubated with mouse anti-IDO (1:200) (Chemicon) and rabbit anti-tryptophan hydroxylase (TPH)2 (1:200) (Cell Signaling Technology, Danvers, MA, USA) antibodies overnight at 4°C. After rinsing with PBS, the sections were incubated with corresponding Alexa Fluor 488 anti-mouse IgG (1:250) and Alexa Fluor 594 anti-rabbit IgG (1:250) (Thermo Fisher Scientific) under dark conditions and room temperature for 60 min. The fluorescence signals from these sections were acquired using a confocal fluorescence microscope (Leica).

### HPLC

#### IDO activity

IDO activity was measured according to a previous study [14]. Mice were sacrificed under deep anesthesia, and striatum tissues were stocked at -80°C. The samples were homogenized with 20 mM phosphate buffer (pH 6.5) containing 0.14 M KCl. After centrifugation, 800 μL of the reaction solution, containing 400 μM tryptophan, 20 mM ascorbic acid, and 10 μM methylene blue in 50 mM phosphate buffer (pH 7.0), was added to 200 μL of supernatant. The samples were then mixed with 100 μg catalase and agitated at 130 rpm/min, 37°C for 6 h. Trichloro acetate (30%) was then added, and the samples were further incubated at 50°C for 30 min. After filtration, the level of kynurenine converted from tryptophan by IDO was measured using the LaChrom Elite HPLC system (Hitachi, Tokyo, Japan). A mixture of 20 mM potassium phosphate (pH 2.5) and acetonitrile (98:2) was used as the mobile phase, and the flow rate of samples was 1 mL/min. The kynurenine peak was detected at 360 nm. IDO activity is represented by the ratio to the control of each trial day using kynurenine level as an index.

#### Serotonin level

The mice were deeply anesthetized 14 days after ICH, a time at which the hematoma was completely absorbed, and blood was removed by perfusion with 10 mL PBS. The ipsilateral brain was dissected out, and the synaptosome fractions were obtained using Syn-PER Synaptic Protein Extraction Reagent (Thermo Fisher Scientific) according to manufacturer instructions. The 5-HT level in the buffer was measured by HPLC (Hitachi). A mixture of 20 mM potassium phosphate (pH 2.5) and acetonitrile (99:1) was used as the mobile phase, and the flow rate of samples was 1 mL/min. The 5-HT peak was detected at 280 nm excitation/340 nm emission fluorescence.

### Behavioral tests

#### Forced swimming test

Behavioral tests were performed independently. A test cage was filled with water to a depth where the mice’s limbs could not touch the bottom. Escape behavior was recorded for 6 min after transferring mice from the home cage to the test cage. The forced swimming test was performed daily from the day after the collagenase injection to day 14. Immobility times recorded in the last 5 of the 6 min on days 1, 3, 7, and 14 after collagenase injection were blindly evaluated as an index of depression-like behavior.

#### Tail suspension test

Escape behavior was recorded when the mice were hung at a height of 50 cm by fixation of their tail. As with the forced swimming test, immobility times were recorded and blindly evaluated.

### Statistics

Data are expressed as means ± SEM. Significant differences were evaluated by the unpaired *t*-test or one-way ANOVA followed by the Student-Newman-Keuls multiple comparison test, unless otherwise indicated, using GraphPad Prism 5. Probability values less than 5% were considered significant. Sample size was estimated with G-power analysis (effect size = 0.5–0.8, α error = 0.05, statistical power = 0.8).

## Results

### IDO expression increased after ICH

**Fig 1:**
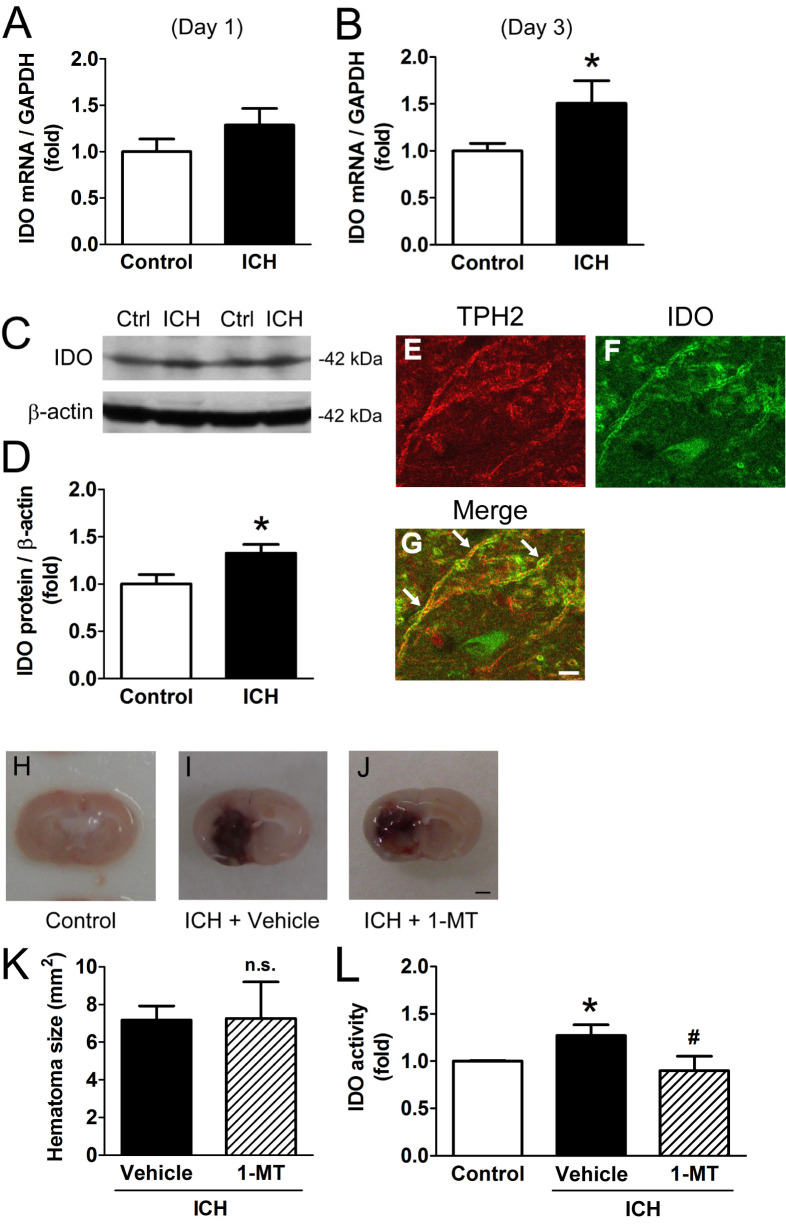
IDO expression increased after ICH. (A) IDO mRNA level 1 day after ICH. Control animals were subjected to intrastriatal injection of saline instead of collagenase (control: n = 12, ICH: n = 10). (B) IDO mRNA level 3 days after ICH; *p < 0.05 vs. control (control: n = 13, ICH: n = 17). (C) Representative blots of IDO and β-actin 3 days after ICH. (D) Changes in IDO/β-actin protein level; *p < 0.05 vs. control (control: n = 11, ICH: n = 10). (E-G) Confocal fluorescence images of TPH2 (red), IDO (green), and merged image. Arrows indicate merged signals. Scale bar = 10 μm. (H-J) Representative images of a coronal section containing a syringe mask 3 days after saline or collagenase injection. Scale bar = 1 mm. (K) Summary of hematoma size; n.s. = not significant; n = 5. (L) IDO activity 3 days after ICH and the effect of 1-MT. IDO activity is represented as the ratio of kynurenine converted from tryptophan to control. Control animals were subjected to intrastriatal injection of saline instead of collagenase and subcutaneous injection of vehicle (saline) instead of 1-MT; *p < 0.05 vs. control, ^#^*p* < 0.05 vs. ICH (control: n = 10, ICH+vehicle: n = 10, ICH+1-MT: n = 6).

First, we investigated the effect of ICH on IDO expression. IDO mRNA level increased 3 days, but not 1 day, after ICH (Fig 1A and 1B). IDO protein level also increased 3 days after ICH (Fig 1C and 1D). Signals of the increased IDO showed co-localization with at least TPH2-positive 5-HTergic neurons (Fig 1E–1G and S1 Fig). Finally, we confirmed the increased level of enzyme protein had normal activity that could be blocked by 1-MT without affecting hematoma size (Fig 1H–1L).

### IDO remains high in the chronic phase and decreases the 5-HT level

**Fig 2:**
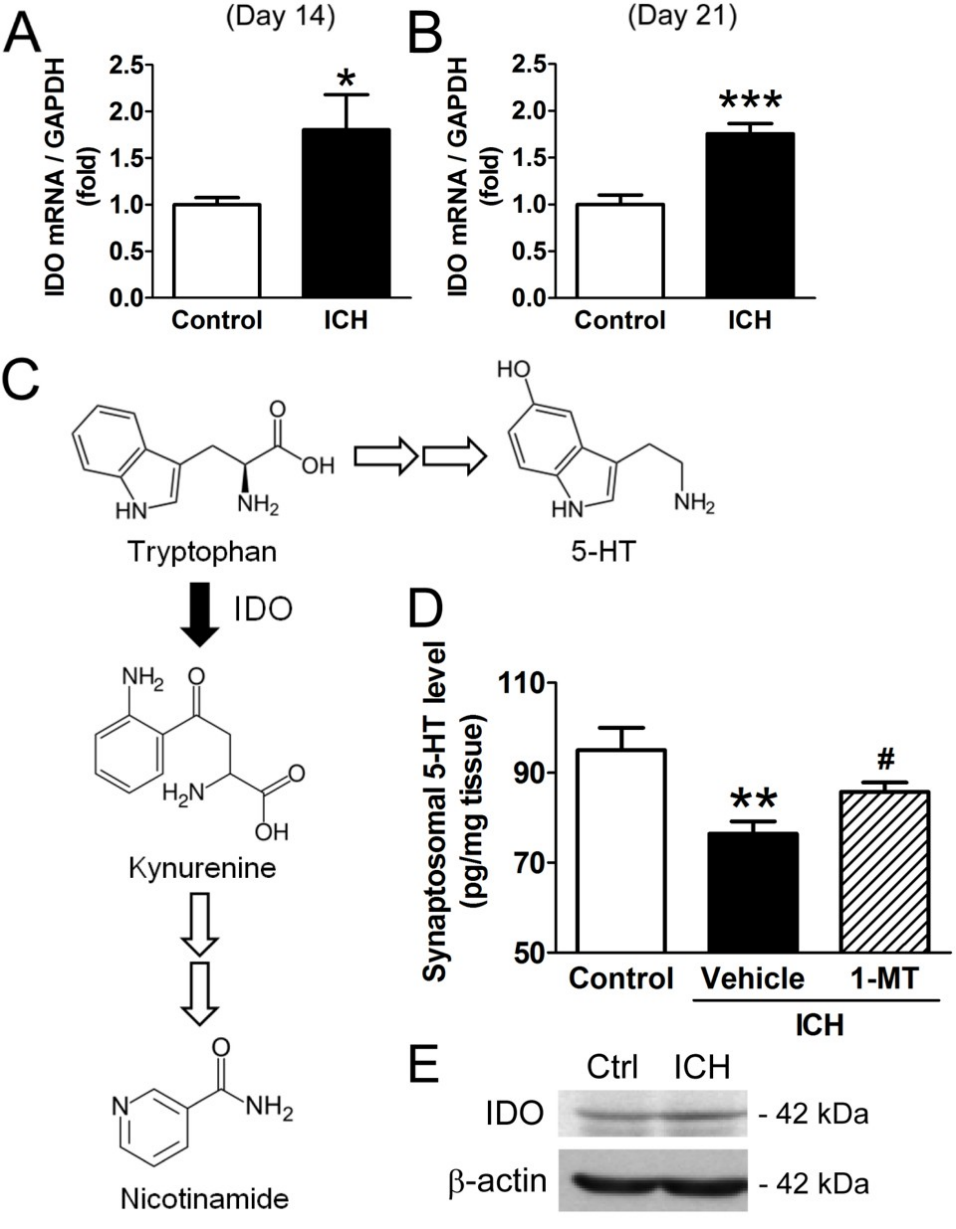
Effect of IDO on brain 5-HT level. (A) IDO mRNA level 14 days after ICH; *p < 0.05 vs. control (control: n = 6, ICH: n = 5). (B) IDO mRNA level 21 days after ICH; ***p < 0.001 vs. control (n = 5 for each group). (C) Scheme of the kynurenine pathway. (D) The effect of 1-MT on synaptosomal 5-HT level 14 days after ICH; **p < 0.01 vs. control, ^#^*p* < 0.05 vs. ICH (control: n = 8, ICH+vehicle: n = 15, ICH+1-MT: n = 10). (E) Representative blots of IDO and β-actin 14 days after ICH.

High IDO mRNA levels were sustained at days 14 and 21 (Fig 2A and 2B). An overview of the kynurenine pathway is shown in Fig 2C. Since we found that increased IDO mRNA level leads to increased IDO activity, we measured the 5-HT level in the synaptosome fraction 14 days after ICH. We found that 5-HT level decreased after ICH, but the administration of 1-MT reversed this effect (Fig 2D), suggesting that the increase of IDO after ICH contributes to the decreased 5-HT level in murine brains. The IDO protein level showed the tendency to increase 14 days after ICH (Fig 2E).

### ICH-induced IDO causes low stress tolerance

**Fig 3:**
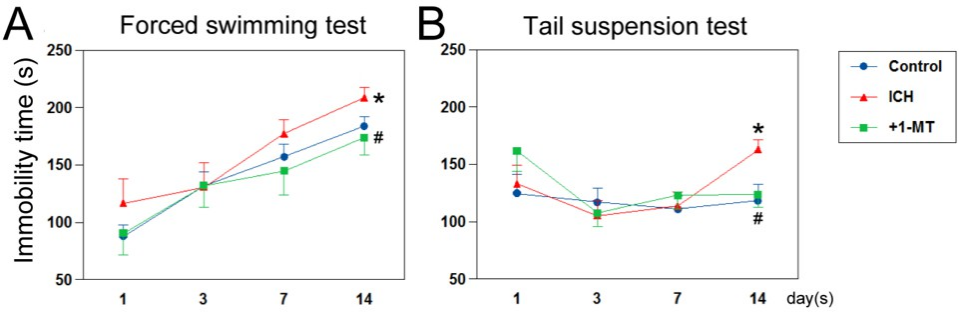
Effect of 1-MT on depression-like behavior after ICH. (A) The effect of 1-MT on immobility time in the forced swimming test after ICH; *p < 0.05 vs. control, ^#^*p* < 0.05 vs. ICH (n = 12–13). Data were analyzed by repeated measures ANOVA. (B) The effect of 1-MT on immobility time in the tail suspension test after ICH; *p < 0.05 vs. control, ^#^*p* < 0.05 vs. ICH at day 14 (n = 14–17).

To examine the possible involvement of the increased IDO on the lowering of motivation after ICH, forced swimming and tail suspension tests were performed. The immobility time in the forced swimming test was prolonged after ICH and that in the tail suspension test was prolonged 14 days after ICH. The administration of 1-MT suppressed both prolongations (Fig 3).

## Discussion

In this study, we found an increase in IDO expression after ICH. IDO mRNA level increased 3 days after ICH, which is similar to a previous report that examined the time course of IDO mRNA levels in hippocampal neurons after transient ischemia [9]. IDO protein level and activity also increased 3 days after ICH, suggesting that mRNA was translated into functional protein. This activity was reversible by the subcutaneous injection of 1-MT without modifying the lesion extent by collagenase.

IDO converts tryptophan to kynurenine in the kynurenine pathway and is may also metabolize 5-HT itself [2, 3]. IDO has been reported to be expressed in neurons [7, 8]; however, whether IDO is expressed in 5-HTergic neurons has not been established. Here, we showed by immunohistochemistry that IDO localizes with TPH2-positive 5-HTergic neurons in the striatum 3 days after ICH. The striatum is the most common site of ICH. Furthermore, in addition to the prefrontal cortex and hippocampus, the striatum is the site in which 5-HTergic neurons have been reported to play a role in depression [15, 16]. We also investigated changes in IDO mRNA levels in the prefrontal cortex and hippocampus, but no significant changes were found in our striatal hemorrhage model (data not shown).

In addition to ischemic stroke, Alzheimer’s disease is known to often be associated with secondary depression [17, 18]. IDO has been reported to increase in disorders, such as Alzheimer’s disease, along with experimental autoimmune encephalomyelitis and multiple sclerosis [17, 19, 20]. These findings support the relationship between depression and IDO. Additionally, lipopolysaccharide (LPS)-induced neuroinflammation has been demonstrated to evoke depression-like behavior through an increase of IDO [21, 22]. Improvement of sucrose preference (%), an index of depression behavior, in LPS-injected mice by IDO-knock out has been previously demonstrated, and the role of IDO has been classified [21]. Apart from IFN, some proinflammatory cytokines, such as tumor necrosis factor (TNF)-α, can stimulate IDO activity [23]. Moreover, it is considered that TNF-α increases after ICH [24]. To clarify the role of IDO in the depression aftereffect of ICH, we measured IDO mRNA and 5-HT levels in the chronic phase of ICH. IDO mRNA levels increased 14 and 21 days after ICH. The synaptosomal 5-HT level decreased 14 days after ICH, and this effect was suppressed by 1-MT administration. To avoid platelet effects, synaptosome fractions after transcardial reflux were analyzed 14 days after ICH, a time at which the hematoma is completely absorbed. Overall, the results suggest that IDO enzyme activity is present 14 days after ICH and is involved in the decrease in 5-HT level during the chronic phase.

Finally, to clarify whether the IDO-induced decrease of 5-HT level contributes to the depression-like behavior after ICH, tail suspension and forced swimming tests were performed. The immobility times of both tests were prolonged after ICH, but recovered by 1-MT administration. It has been suggested that the changes to the brain network occurs gradually as the level of 5-HT increases [25, 26]. This gradual change may be related to our observation that depressive symptoms improved after 14 days. To confirm whether or not the prolongation of immobility times was due to motor dysfunction caused by ICH, an open field test was performed. Locomotor activity did not decrease after ICH (S2 Fig). This finding is consistent with a previous report showing increased locomotor activity in ischemic gerbils [27]. Taken together, IDO increased after ICH, resulting in depression-like behavior due to the reduced stress tolerance accompanying the decrease in 5-HT level. Although variations in IDO activity and 5-HT synaptosomal levels after 1-MT treatment were slight, the behavior of animals certainly improved. Some reports have shown that recovery of the 5-HT level equivalent to that in this study leads to the improvement of immobility times of forced swimming and tail suspension tests in the murine lipopolysaccharide-induced depression model, suggesting that their slight variation has the significance [28, 29]. There was absence of nitric oxide (NO) production and extremely high levels of IDO in human monocytic cells, whereas murine cells expressed abundant inducible NO synthase and very little IDO [30, 31]. Thus, IDO may play a more important role in humans. These findings suggest that IDO is a novel therapeutic target for the decreased motivation after ICH.

## Conclusion

For the first time, we have shown that IDO in 5-HT neurons is up-regulated after ICH. This up-regulation decreased the 5-HT levels in the brain, causing low stress tolerance after ICH. The results of this study suggest that IDO is a novel therapeutic target for the depression aftereffect of ICH.

## Supporting information

S1 Fig Original images of immunofluorescence of TPH2, IDO, and merged images in Fig 1E–1G. Scale bar = 20 μm.
(TIF)

S2 Fig Locomotive activity in the open field test after ICH. Mice were transferred to a test cage (260 mm by 420 mm, 20 mm high). The test cage floor was lined at 50 mm by 50 mm intervals. Mice were allowed to explore the new environment, were video-recorded for 5 min, and the frequency of crossing the lines was counted. The data were analyzed by unpaired *t*-test with Welch’s correction. n = 6–8.
(TIF)
