# Supplementary figures and images for: Indoleamine 2, 3-dioxygenase is responsible for low stress tolerance after intracerebral hemorrhage
Source: PLoS One. 2023 Feb 8;18(2):e0273037. doi: 10.1371/journal.pone.0273037 (PMC9907831; doi:10.1371/journal.pone.0273037)

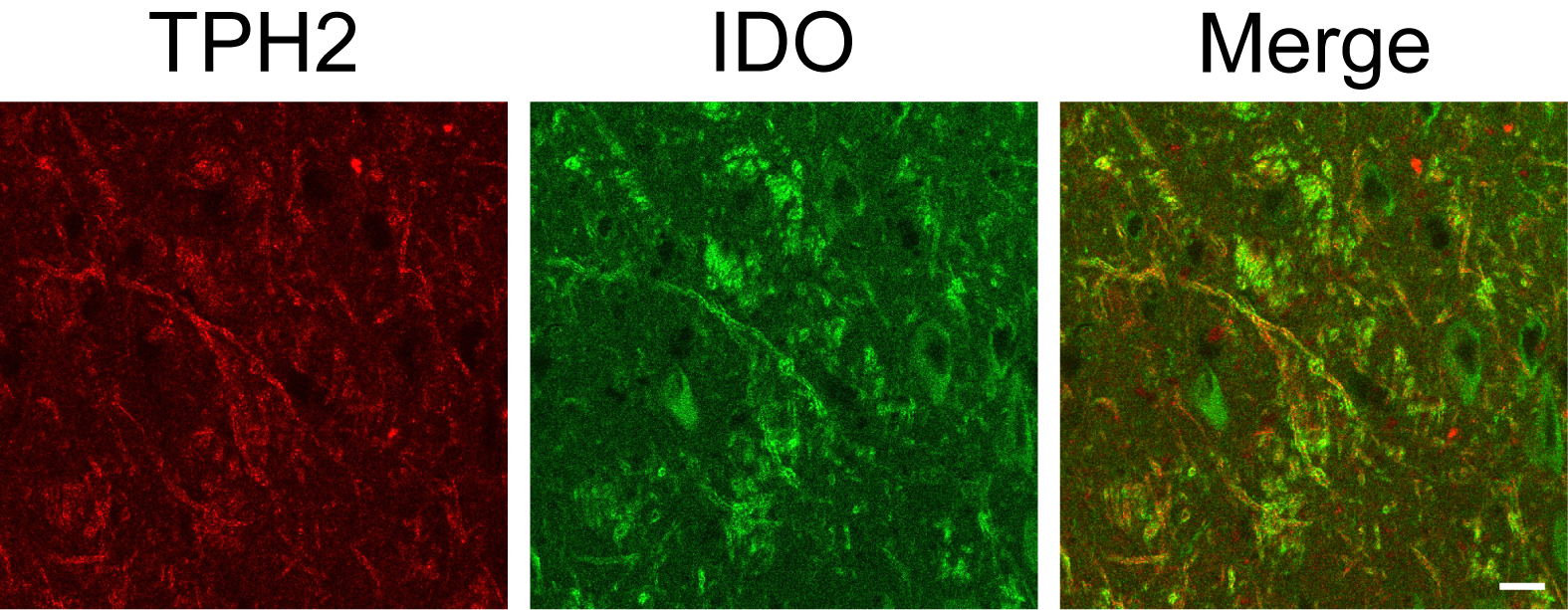

Supplement: S1 Fig — Scale bar = 20 μm. (TIF) [file pone.0273037.s001.tif]

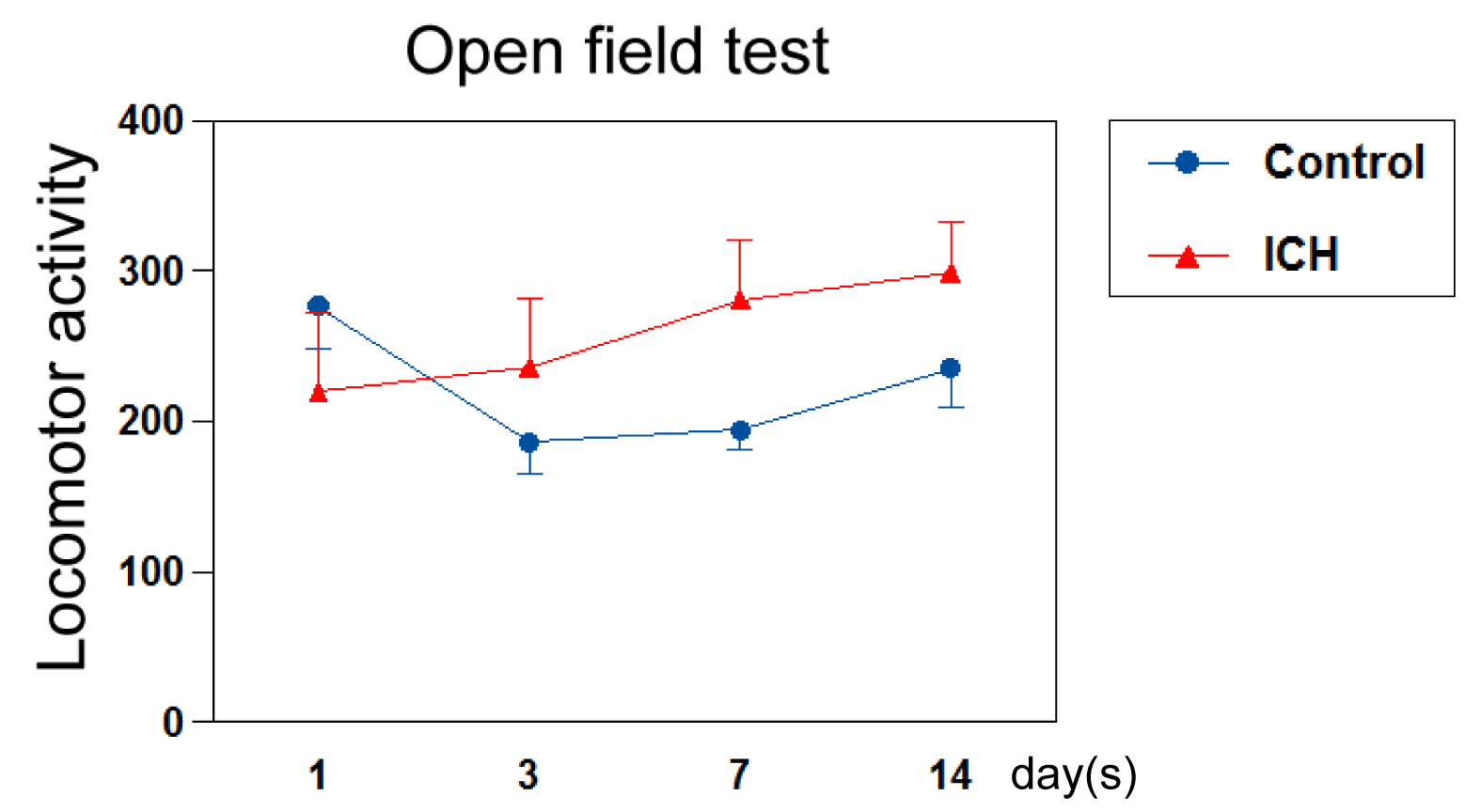

Supplement: S2 Fig — Mice were transferred to a test cage (260 mm by 420 mm, 20 mm high). The test cage floor was lined at 50 mm by 50 mm intervals. Mice were allowed to explore the new environment, were video-recorded for 5 min, and the frequency of crossing the lines was counted. The data were analyzed by unpaired t-test with Welch’s correction. n = 6–8. (TIF) [file pone.0273037.s002.tif]
